# 2,4-Dichloro-6-(3-methyl­piperidin-1-yl)-1,3,5-triazine

**DOI:** 10.1107/S1600536808013214

**Published:** 2008-06-21

**Authors:** Wei Wang

**Affiliations:** a Ordered Matter Science Research Center, Southeast University, Nanjing 210096, People’s Republic of China

## Abstract

In the title compound, C_9_H_12_Cl_2_N_4_, the piperidine ring adopts a chair conformation. The electron delocalization of the molecule is indicated by the similar C⋯N distances within the triazine ring and by the double-bond character of the C=N triazine–piperidine connectivity. Weak intra­molecular C—H⋯N hydrogen bonds link the two rings within the mol­ecule, which exhibits a pseudo-mirror plane if the methyl group is ignored. π–π Inter­actions between pairs of triazine rings with stacking distances of 3.521 (7) Å are observed in the crystal structure, generated *via* crystallographic inversion centers.

## Related literature

For general background and the experimental method, see: Sandford (2003 ▶); Masllorens *et al.* (2004 ▶); Ciunik (1997 ▶); Hunter & Sanders (1990 ▶); Taylor & Kennard (1982 ▶); Thalladi *et al.* (1998 ▶).
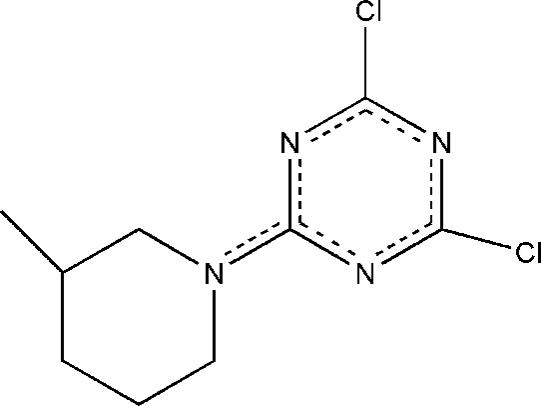


## Experimental

### 

#### Crystal data

C_9_H_12_Cl_2_N_4_
*M* *_r_* = 247.13
Monoclinic, 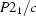

*a* = 8.086 (16) Å
*b* = 19.19 (3) Å
*c* = 7.813 (15) Å
β = 106.18 (3)°
*V* = 1164 (4) Å^3^
*Z* = 4
Mo *K*α radiation
μ = 0.53 mm^−1^
*T* = 293 (2) K
0.40 × 0.20 × 0.15 mm

#### Data collection

Rigaku Mercury2 diffractometer
Absorption correction: multi-scan (*CrystalClear*; Rigaku, 2005 ▶) *T*_min_ = 0.750, *T*_max_ = 1.000 (expected range = 0.692–0.923)
11851 measured reflections
2765 independent reflections
1118 reflections with *I* > 2σ(*I*)
*R*_int_ = 0.068

#### Refinement

*R*[*F* ^2^ > 2σ(*F* ^2^)] = 0.058
*wR*(*F* ^2^) = 0.189
*S* = 0.86
2765 reflections
136 parameters
H-atom parameters constrained
Δρ_max_ = 0.35 e Å^−3^
Δρ_min_ = −0.27 e Å^−3^

### 

Data collection: *CrystalClear* (Rigaku, 2005 ▶); cell refinement: *CrystalClear*; data reduction: *CrystalClear*; program(s) used to solve structure: *SHELXS97* (Sheldrick, 2008 ▶); program(s) used to refine structure: *SHELXL97* (Sheldrick, 2008 ▶); molecular graphics: *SHELXTL* (Sheldrick, 2008 ▶); software used to prepare material for publication: *SHELXTL*.

## Supplementary Material

Crystal structure: contains datablocks I, global. DOI: 10.1107/S1600536808013214/si2085sup1.cif

Structure factors: contains datablocks I. DOI: 10.1107/S1600536808013214/si2085Isup2.hkl

Additional supplementary materials:  crystallographic information; 3D view; checkCIF report

## Figures and Tables

**Table 1:** Hydrogen-bond geometry (Å, °)

*D*—H⋯*A*	*D*—H	H⋯*A*	*D*⋯*A*	*D*—H⋯*A*
C5—H5*B*⋯N4	0.97	2.34	2.787 (6)	108
C9—H9*B*⋯N2	0.97	2.35	2.794 (6)	107

**Fig. 1.**
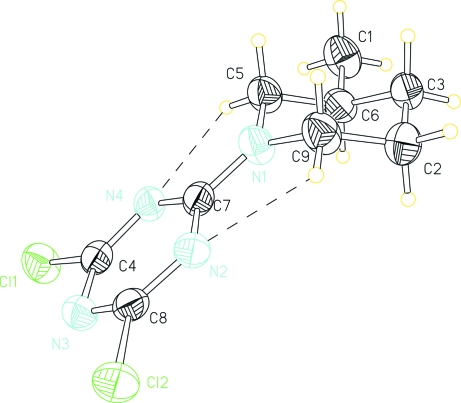
View of the molecular structure of the title compound with the atomic numbering scheme. Displacement ellipsoids were drawn at the 30% probability level.

**Fig. 2.**
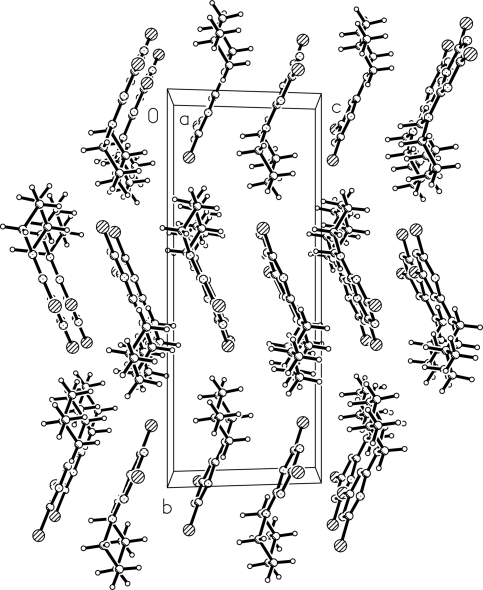
The packing diagram of the title compound, viewed along the a axis.

## supplementary crystallographic information

### Comment

2,4,6-Trichloro-1,3,5-triazine is an interesting building block since it shows an unusual ability of replacement of the chlorine atoms by nucleophiles. It is often used for the construction of an array of novel complex derivatives and of a variety of structurally diverse macrocycles by sequential nucleophilic aromatic substitution processes (Sandford, 2003; Masllorens *et al*., 2004). Besides, it can also be used to construct a target supramolecular network. A series of substituted triazine compounds stabilized by weak intermolecular interactions such as C—H···N hydrogen bonding and π···π interaction were reported before (Thalladi *et al.*, 1998). Crystallographic evidence for the existence of C—H···N hydrogen bonds with H···N ranges between 2.52 and 2.72 Å was communicated by Taylor & Kennard (1982).

In the title compound, C_9_H_12_Cl_2_N_4_, the methylpiperidine group adopts a chair conformation and the chiral C6 atom is in S^*^ configuration (Figure 1). If the methyl group at the piperidine group is replaced by a hydrogen atom, the molecule is nearly mirror symmetrical. The crystal data shows that the N—C bond lengths of N1—C7, N3—C4 and N3—C8 are 1.330 (5), 1.344 (5) and 1.340 (5) Å respectively. These relative homogeneous bond distances indicate the inflexibility of the molecule. Though no classic hydrogen bond is found, there is evidence of weak C—H···N interactions in the molecule (Table 1). In contrast to these inflexible intramolecular C—H···N hydrogen bonds, an example of intramolecular C—H···N hydrogen bond interactions showed a stabilizing effect in the conformation of flexible pyranoid rings (Ciunik, 1997).

Fig. 2 shows the packing diagram and the stacking between pairs of pyrazine rings. The stacking distance between the ring centroids *Cg*···*Cg*^i^ is 3.521 (7) Å, indicating quite strong π···π interactions between the symmetry-related molecules (symmetry code: -*x*, 1 - *y*,1 - *z*). This face to face π···π interaction plays a very important function in stabilizing the crystal structure (Hunter & Sanders, 1990).

### Experimental

2,4,6-Trichloro-1,3,5-triazine (1.84 g, 10 mmol) and 3-methylpiperidine (0.99 g, 10 mmol) were dissolved in the mixture of acetone (25 ml) and H_2_O (5 ml) in the presence of KOH (0.56 g, 10 mmol) and refluxed for 24 h. The conversion of reaction was monitored by TLC. After the mixture was cooled to room temperature, the solution was filtered and rotated in vacuum. A white solid was obtained after purification by column chromatography on silica gel (n-18 hexane). Colorless crystals suitable for single-crystal X-ray diffraction studies were obtained by slow evaporation of a solution in ethanol at room temperature over several days.

### Refinement

Positional parameters of all the H atoms were calculated geometrically and were allowed to ride on the C atoms to which they are bonded, with *U*_iso_(H) = 1.2*U*_eq_(C).

### Crystal data

**Table:**

C_9_H_12_Cl_2_N_4_	*F*_000_ = 512
*M**_r_* = 247.13	*D*_x_ = 1.409 Mg m^−^^3^
Monoclinic, *P*2_1_/*c*	Mo *K*α radiation λ = 0.71073 Å
Hall symbol: -P 2ybc	Cell parameters from 2183 reflections
*a* = 8.086 (16) Å	θ = 3.4–27.4º
*b* = 19.19 (3) Å	µ = 0.53 mm^−^^1^
*c* = 7.813 (15) Å	*T* = 293 (2) K
β = 106.18 (3)º	Block, colorless
*V* = 1164 (4) Å^3^	0.40 × 0.20 × 0.15 mm
*Z* = 4	

### Data collection

**Table:**

Rigaku Mercury2 diffractometer	2765 independent reflections
Radiation source: fine-focus sealed tube	1118 reflections with *I* > 2σ(*I*)
Monochromator: graphite	*R*_int_ = 0.068
Detector resolution: 13.6612 pixels mm^-1^	θ_max_ = 27.9º
*T* = 293(2) K	θ_min_ = 2.6º
CCD_Profile_fitting scans	*h* = −10→10
Absorption correction: multi-scan(CrystalClear; Rigaku, 2005)	*k* = −25→25
*T*_min_ = 0.750, *T*_max_ = 1.000	*l* = −10→10
11851 measured reflections	

### Refinement

**Table:**

Refinement on *F*^2^	Secondary atom site location: difference Fourier map
Least-squares matrix: full	Hydrogen site location: inferred from neighbouring sites
*R*[*F*^2^ > 2σ(*F*^2^)] = 0.058	H-atom parameters constrained
*wR*(*F*^2^) = 0.189	*w* = 1/[σ^2^(*F*_o_^2^) + (0.09*P*)^2^] where *P* = (*F*_o_^2^ + 2*F*_c_^2^)/3
*S* = 0.86	(Δ/σ)_max_ < 0.001
2765 reflections	Δρ_max_ = 0.35 e Å^−^^3^
136 parameters	Δρ_min_ = −0.27 e Å^−^^3^
Primary atom site location: structure-invariant direct methods	Extinction correction: none

### Special details

**Table:**

Experimental. The relative large standard uncertainties (s. u.) noted in Alert level B of PLATON may be explained by measurement at room temperature and weak diffraction power of the crystal.
Geometry. All e.s.d.'s (except the e.s.d. in the dihedral angle between two l.s. planes) are estimated using the full covariance matrix. The cell e.s.d.'s are taken into account individually in the estimation of e.s.d.'s in distances, angles and torsion angles; correlations between e.s.d.'s in cell parameters are only used when they are defined by crystal symmetry. An approximate (isotropic) treatment of cell e.s.d.'s is used for estimating e.s.d.'s involving l.s. planes.
Refinement. Refinement of *F*^2^ against ALL reflections. The weighted *R*-factor *wR* and goodness of fit *S* are based on *F*^2^, conventional *R*-factors *R* are based on *F*, with *F* set to zero for negative *F*^2^. The threshold expression of *F*^2^ > 2σ(*F*^2^) is used only for calculating *R*-factors(gt) *etc*. and is not relevant to the choice of reflections for refinement. *R*-factors based on *F*^2^ are statistically about twice as large as those based on *F*, and *R*- factors based on ALL data will be even larger.

### Fractional atomic coordinates and isotropic or equivalent isotropic displacement parameters (Å^2^)

**Table:**

	*x*	*y*	*z*	*U*_iso_*/*U*_eq_	
Cl2	0.33512 (12)	0.54293 (6)	0.33576 (14)	0.0794 (4)	
Cl1	−0.24201 (14)	0.64892 (6)	0.38583 (16)	0.0890 (4)	
N4	−0.2234 (3)	0.52641 (15)	0.2549 (4)	0.0597 (8)	
N3	0.0402 (4)	0.59106 (15)	0.3557 (4)	0.0641 (8)	
N2	0.0437 (4)	0.47725 (15)	0.2313 (4)	0.0615 (8)	
C9	−0.1242 (5)	0.35428 (19)	0.0892 (6)	0.0774 (12)	
H9A	−0.1644	0.3467	−0.0385	0.093*	
H9B	−0.0010	0.3627	0.1210	0.093*	
C8	0.1142 (4)	0.53582 (19)	0.3024 (4)	0.0598 (9)	
N1	−0.2137 (4)	0.41552 (15)	0.1395 (4)	0.0682 (9)	
C7	−0.1306 (4)	0.47334 (18)	0.2081 (5)	0.0574 (9)	
C6	−0.4391 (5)	0.34335 (18)	0.2078 (5)	0.0681 (10)	
H6A	−0.3900	0.3513	0.3359	0.082*	
C5	−0.4023 (4)	0.4065 (2)	0.1093 (6)	0.0711 (11)	
H5A	−0.4575	0.4012	−0.0172	0.085*	
H5B	−0.4496	0.4477	0.1502	0.085*	
C4	−0.1300 (5)	0.58034 (19)	0.3233 (5)	0.0600 (9)	
C3	−0.3548 (5)	0.27924 (19)	0.1560 (5)	0.0720 (11)	
H3A	−0.3735	0.2397	0.2257	0.086*	
H3B	−0.4074	0.2688	0.0312	0.086*	
C2	−0.1620 (5)	0.2904 (2)	0.1872 (6)	0.0826 (12)	
H2A	−0.1078	0.2958	0.3139	0.099*	
H2B	−0.1127	0.2496	0.1470	0.099*	
C1	−0.6342 (6)	0.3354 (2)	0.1702 (7)	0.0919 (14)	
H1A	−0.6814	0.3769	0.2065	0.138*	
H1B	−0.6590	0.2962	0.2356	0.138*	
H1C	−0.6846	0.3279	0.0450	0.138*	

### Atomic displacement parameters (Å^2^)

**Table:**

	*U*^11^	*U*^22^	*U*^33^	*U*^12^	*U*^13^	*U*^23^
Cl2	0.0528 (6)	0.0896 (8)	0.0908 (8)	−0.0078 (5)	0.0118 (5)	0.0008 (6)
Cl1	0.0794 (8)	0.0676 (7)	0.1162 (10)	0.0117 (5)	0.0206 (7)	−0.0158 (6)
N4	0.0487 (16)	0.0527 (17)	0.075 (2)	0.0032 (14)	0.0119 (15)	0.0052 (15)
N3	0.0560 (19)	0.0611 (19)	0.072 (2)	−0.0043 (15)	0.0119 (15)	−0.0010 (15)
N2	0.0494 (16)	0.0615 (18)	0.0692 (19)	−0.0032 (14)	0.0092 (14)	0.0042 (15)
C9	0.064 (2)	0.063 (2)	0.102 (3)	0.004 (2)	0.018 (2)	−0.014 (2)
C8	0.0500 (19)	0.067 (2)	0.056 (2)	−0.0029 (18)	0.0052 (17)	0.0127 (18)
N1	0.0432 (16)	0.0593 (19)	0.100 (2)	−0.0032 (14)	0.0157 (16)	−0.0056 (17)
C7	0.0513 (19)	0.053 (2)	0.064 (2)	0.0009 (17)	0.0095 (17)	0.0117 (17)
C6	0.074 (3)	0.060 (2)	0.070 (2)	−0.0073 (19)	0.018 (2)	−0.0074 (19)
C5	0.052 (2)	0.062 (2)	0.094 (3)	−0.0047 (18)	0.012 (2)	−0.002 (2)
C4	0.062 (2)	0.056 (2)	0.059 (2)	0.0055 (18)	0.0129 (18)	0.0089 (17)
C3	0.090 (3)	0.054 (2)	0.070 (3)	−0.002 (2)	0.019 (2)	−0.0021 (18)
C2	0.085 (3)	0.066 (3)	0.092 (3)	0.015 (2)	0.018 (2)	−0.001 (2)
C1	0.077 (3)	0.081 (3)	0.125 (4)	−0.012 (2)	0.041 (3)	−0.010 (3)

### Geometric parameters (Å, °)

**Table:**

Cl2—C8	1.737 (5)	C6—C5	1.510 (5)
Cl1—C4	1.743 (4)	C6—C1	1.529 (6)
N4—C4	1.305 (5)	C6—C3	1.516 (5)
N4—C7	1.373 (4)	C6—H6A	0.9800
N3—C8	1.340 (5)	C5—H5A	0.9700
N3—C4	1.344 (5)	C5—H5B	0.9700
N2—C8	1.312 (5)	C3—C2	1.524 (6)
N2—C7	1.372 (5)	C3—H3A	0.9700
C9—C2	1.521 (6)	C3—H3B	0.9700
C9—N1	1.489 (5)	C2—H2A	0.9700
C9—H9A	0.9700	C2—H2B	0.9700
C9—H9B	0.9700	C1—H1A	0.9600
N1—C7	1.330 (5)	C1—H1B	0.9600
N1—C5	1.487 (5)	C1—H1C	0.9600
			
C4—N4—C7	113.6 (3)	C6—C5—H5A	109.5
C8—N3—C4	110.1 (3)	N1—C5—H5B	109.5
C8—N2—C7	114.3 (3)	C6—C5—H5B	109.5
C2—C9—N1	108.8 (3)	H5A—C5—H5B	108.1
C2—C9—H9A	109.9	N4—C4—N3	130.1 (3)
N1—C9—H9A	109.9	N4—C4—Cl1	115.3 (3)
C2—C9—H9B	109.9	N3—C4—Cl1	114.6 (3)
N1—C9—H9B	109.9	C2—C3—C6	111.1 (3)
H9A—C9—H9B	108.3	C2—C3—H3A	109.4
N2—C8—N3	129.1 (3)	C6—C3—H3A	109.4
N2—C8—Cl2	116.0 (3)	C2—C3—H3B	109.4
N3—C8—Cl2	114.9 (3)	C6—C3—H3B	109.4
C7—N1—C5	122.8 (3)	H3A—C3—H3B	108.0
C7—N1—C9	122.5 (3)	C9—C2—C3	111.9 (3)
C5—N1—C9	114.8 (3)	C9—C2—H2A	109.2
N1—C7—N2	118.9 (3)	C3—C2—H2A	109.2
N1—C7—N4	118.3 (3)	C9—C2—H2B	109.2
N2—C7—N4	122.8 (3)	C3—C2—H2B	109.2
C5—C6—C1	108.8 (3)	H2A—C2—H2B	107.9
C5—C6—C3	110.4 (3)	C6—C1—H1A	109.5
C1—C6—C3	112.6 (3)	C6—C1—H1B	109.5
C5—C6—H6A	108.3	H1A—C1—H1B	109.5
C1—C6—H6A	108.3	C6—C1—H1C	109.5
C3—C6—H6A	108.3	H1A—C1—H1C	109.5
N1—C5—C6	110.6 (3)	H1B—C1—H1C	109.5
N1—C5—H5A	109.5		
			
C7—N2—C8—N3	0.6 (5)	C4—N4—C7—N2	−0.1 (5)
C7—N2—C8—Cl2	179.6 (2)	C7—N1—C5—C6	−122.5 (4)
C4—N3—C8—N2	−1.3 (5)	C9—N1—C5—C6	56.7 (4)
C4—N3—C8—Cl2	179.7 (2)	C1—C6—C5—N1	−178.9 (3)
C2—C9—N1—C7	124.0 (4)	C3—C6—C5—N1	−54.9 (4)
C2—C9—N1—C5	−55.2 (4)	C7—N4—C4—N3	−0.8 (5)
C5—N1—C7—N2	−179.9 (3)	C7—N4—C4—Cl1	−179.7 (2)
C9—N1—C7—N2	0.9 (5)	C8—N3—C4—N4	1.4 (5)
C5—N1—C7—N4	0.8 (5)	C8—N3—C4—Cl1	−179.7 (2)
C9—N1—C7—N4	−178.3 (3)	C5—C6—C3—C2	55.6 (4)
C8—N2—C7—N1	−179.0 (3)	C1—C6—C3—C2	177.3 (3)
C8—N2—C7—N4	0.2 (5)	N1—C9—C2—C3	53.9 (4)
C4—N4—C7—N1	179.0 (3)	C6—C3—C2—C9	−55.9 (5)

### Hydrogen-bond geometry (Å, °)

**Table:**

*D*—H···*A*	*D*—H	H···*A*	*D*···*A*	*D*—H···*A*
C5—H5B···N4	0.97	2.34	2.787 (6)	108
C9—H9B···N2	0.97	2.35	2.794 (6)	107
